# Successful treatment of pulmonary embolism in a patient with polycythemia vera by rheolytic thrombectomy

**DOI:** 10.15171/jcvtr.2019.54

**Published:** 2019-05-01

**Authors:** Tufan Çinar, Mert İlker Hayıroğlu, Ahmet Oz

**Affiliations:** Health Sciences University, Sultan Abdülhamid Han Training and Research Hospital, Department of Cardiology, Istanbul, Turkey

**Keywords:** Acute Pulmonary Embolism, Polycythemia Vera, Rheolytic Thrombectomy, Contraindication

## Abstract

Acute pulmonary embolism (APE) may lead to life-threatening conditions such as cardiac death and congestive heart failure. Thus, a proper diagnosis and management play a crucial role to prevent such complications. Moreover, APE is a rare clinical onset of chronic myeloproliferative disease. We herein describe a 67-year-old patient with polycythemia vera presented to our cardiology clinic with pulmonary embolism despite the fact that an intense antiplatelet treatment started secondary to acute myocardial infarction prior. Because the patient had hypotension and head trauma, rheolytic thrombectomy was performed successfully to restore adequate pulmonary perfusion.

## Introduction

Acute pulmonary embolism (APE) is a rare life-threatening clinical entity occurs more frequently in patients with pro-coagulative conditions. The clinical diagnosis of APE is difficult due to its obscure signs and symptoms. APE is treated with anticoagulation but treatment strategy may change according to the various etiologies and clinical status on the presentation. Here we are presenting an APE case in a patient with polycythemia vera, which was successfully treated with rheolytic thrombectomy due to contraindication of routine thrombolytic therapy.

## Case Report

**Figure 1:**
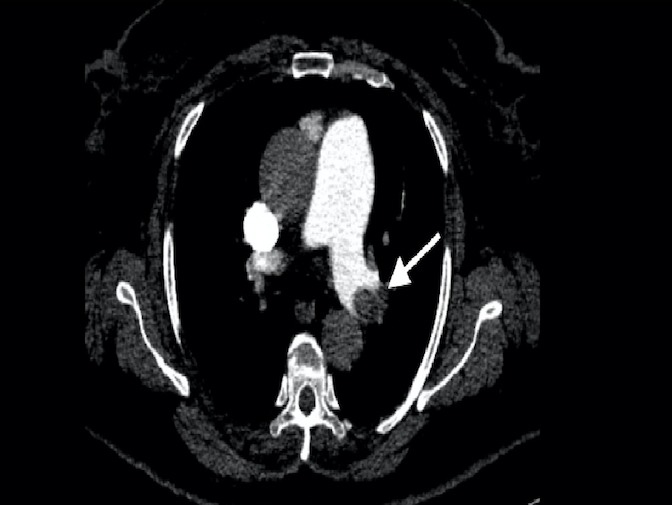
A thrombosis that is found in the left main pulmonary artery provided by contrast enhanced thoracic computed tomography (arrows).

**Figure 2:**
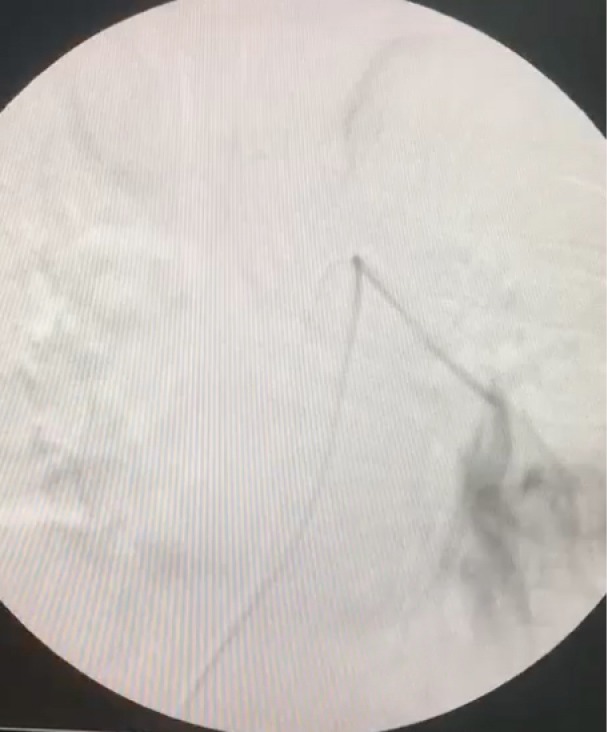
A subtotal thrombosis in the left main pulmonary artery before the rheolytic therapy obtained with a multipurpose catheter.

**Figure 3:**
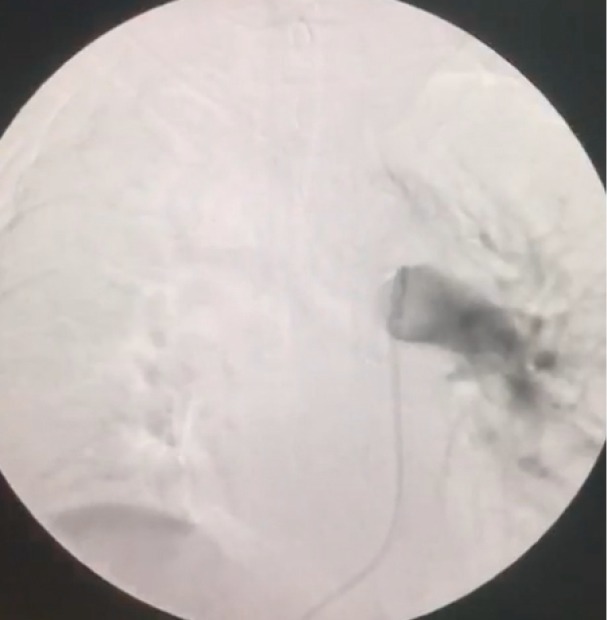
A successful recanalization of the left main pulmonary artery after the rheolytic therapy obtained with a pigtail catheter.

A 67-year-old female patient presented to our cardiology clinic with a chest pain that started eight hours ago. The character of the chest pain was squeezing, located retrosternal, radiating to the left arm. The patient had neither palpitation nor dyspnea. Medical history was significant for hypertension and polycythemia vera that was diagnosed 7 years ago. She had been followed up regularly and had been taking hydroxyurea 300 mg two times a day. The electrocardiogram (ECG) of the patient showed a normal sinus rhythm and no any ischemic finding was present. The blood pressure of the patient was 120/70 mm/Hg. On physical examination, auscultation of the chest revealed no pathological sounds or murmurs. The transthoracic echocardiography (TTE) was performed, providing an ejection fraction of 65%, left ventricular hypertrophy, minimal tricuspid insufficiency. The laboratory results included: hemoglobin level of 17.0 g/dL (13.5–17.0 g/dL), hematocrit of 48.4% (0.39–0.50), D-dimer of 328.3 of FEU/L (0-500), and troponin I of 0.58 ng/mL (0-0.03). The patient was hospitalized with acute coronary syndrome preliminary diagnosis and treated with acetylsalicylic acid 1x100 mg, clopidogrel 4 x 75 mg with a loading dose and maintenance dose with 1x75mg, metoprolol 1x50 mg, ramipril 1 x 5 mg, atorvastatin 1x80 mg, enoxaparin 2 x 0.6 cc. Coronary angiography was performed via the right femoral artery. Critical lesions were found in the diagonal branch of left anterior descending artery (LAD) and right coronary artery (RCA). A 4.0 x 12 mm bare metal stent was implanted to the RCA and a 2.75 x 18 mm drug eluting stent implanted to the diagonal branch of LAD. No complication was observed after the procedure. The patient was discharged well next day under the medical therapy. Three days following the discharge, she was admitted to emergency department with sudden onset chest pain associated with shortness of breath. The patient also experienced syncope at home and had a head trauma. The ECG demonstrated sinus tachycardia. On physical examination, the patient oxygen saturation was 90% and blood pressure was 80/55 mm/Hg. Laboratory results included: hemoglobin level of 18g/dL (13.5–17.0 g/dL), hematocrit of 57.3 % (0.39–0.50), troponin I level of 0.170 ng/mL (0-0.03), and D-dimer of 795.4 FEU/L (0-500). The TTE provided a normal left ventricular systolic function and diameter (49 mm), right ventricular dilatation (42 mm, the ratio of left ventricle to right ventricle was 0.91), reduced right ventricular systolic function (tricuspid annular plane systolic excursion: 1.7 cm), and an estimated pulmonary artery systolic pressure of 55 mmHg. Contrast enhanced pulmonary computed tomographic angiography showed filling defect concordant with thrombus in the left main pulmonary artery (Figure 1). Even though there was a therapeutic alternative with a thrombolytic treatment, we did not consider of it due to past head trauma. Therefore, the decision was made to perform a rheolytic thrombectomy. After the insertion of a 6-F femoral sheath into the right femoral vein, a steerable 0.035’’ inch guidewire was advanced over multipurpose catheter form the inferior vena cava to the main pulmonary artery. Then, a guidewire was positioned in the left main pulmonary artery. Multipurpose catheter was exchanged with the AnjioJet^®^PE catheter (Boston Scientific, USA) (Figure 2). The AnjioJet^®^PE catheter was activated for 20 seconds and slowly advanced over the thrombus and pulled back three or four times for clot removal. During the procedure, unfractionatedheparin was administrated in order to obtain an activated clotting time of 250-300 seconds. Treatment was performed successfully and no complication was observed (Figure 3). Anticoagulation treatment with warfarin was started. The patient was discharged well from the hospital on the sixth day following the level of international normalized ratio (INR) of 2.6.

## Discussion

Polycythemia vera is a clonal stem-cell myeloproliferative disease that is Philadelphia chromosome–negative.^1^ The Janus kinase (JAK 2) mutation accounts for 95% of polycythemia cases.^2^ The increased blood viscosity, which raises primarily from high red blood cells counts, can contribute to the formation of thrombi. Thrombotic complications such as stroke, myocardial infarction, deep vein thrombosis and pulmonary embolism are the primary cause of mortality in patients with polycythemia vera, responsible for 45% of all deaths.^3^ Cytoreductive medications including hydroxyurea, interferon alpha (IFN-α), and busulphan were recommended to control red blood cell volume in patients in whom phlebotomy is poorly tolerated or those in whom the thrombotic risk remains high.^4^ Hydroxyurea, which is a currently the treatment of choice for patients with polycythemia vera who are older than 40 years of age, effectively improves myelosuppression and reduces the risk for thrombosis compared with the use of phlebotomy alone.^5^ In our case report, the patient experienced a thrombotic complication as APE in spite of treatment with hydroxyurea.

APE is a deadly event that results from obstruction of pulmonary artery or one of its branches with a thrombus. APE may remain asymptomatic or its diagnosis may be an incidental finding or the first presentation may even be sudden death.^6^ APE should be suspected in patients with symptoms of chest pain, pre-syncope or syncope, and hemoptysis.^7-9^ Chest pain is usually caused by pleural irritation due to distal emboli causing pulmonary infarction. Due to clinical assessment cannot confirm or exclude APE, increasing data support the use of clinical prediction models as Wells and Revised Geneva scores to guide the diagnostic approach.^10,11^ Echocardiography may help the diagnosis of APE but computed tomographic (CT) angiography is the gold standard imaging in patients with suspected APE.^12^

The main treatment of APE consists of stabilization of the patient, anticoagulation treatment and the use of systemic thrombolytic agent in patients with hypotension or shock. The systemic thrombolytic treatment is also recommended in patients with right ventricular dysfunction (by echocardiography or CT angiography or elevated cardiac troponin).^13^ When systemic thrombolytic agents are contraindicated because of increased of risk of bleeding, as in our case report due to a recent head trauma, catheter-based interventional treatments are recommended. Systemic review and meta-analysis done by Kuo et al on the catheter-based interventional treatments in patients with high risk APE showed a successful resolution of hypoxemia and hemodynamic improvement.^14^ To the best of our knowledge, there are few case reports of acute APE in patients with polycythemia vera. However, this is the first case report presenting successful treatment of APE with a rheolytic thrombectomy in a patient with polycythemia vera in whom thrombolytic treatment was contraindicated due to recent head trauma.

## Conclusion

APE may occur in a patient with polycythemia vera despite the intense antiplatelet treatment which was started due to recent myocardial infarction. It should be remembered that the patients who have a contraindication for thrombolytic treatment the rheolytic thrombectomy can be performed in situations requiring urgent recanalization of the pulmonary arterial system.

## Competing interests

All authors declare that they do not have conflict of interest.

## Ethical approval

Informed consent was obtained from the patient for publishing this case report.
